# Simultaneous strength and ductility enhancements of high thermal conductive Ag7.5Cu alloy by selective laser melting

**DOI:** 10.1038/s41598-022-08182-4

**Published:** 2022-03-11

**Authors:** Wei Xiong, Liang Hao, Ton Peijs, Chunze Yan, Kaka Cheng, Ping Gong, Qian Cui, Danna Tang, Shamoon Al Islam, Yan Li

**Affiliations:** 1 China University of Geosciences, Wuhan, 430074 People’s Republic of China; 2 WMG, Materials Engineering Centre, University of Warwick, Coventry, CV4 7AL UK; 3 School of Materials Science and Engineering, Huazhong University of Science and Technology, Wuhan, 430074 People’s Republic of China; 4 Hubei Gem & Jewelry Engineering Technology Research Center, Wuhan, 430074 People’s Republic of China

**Keywords:** Engineering, Materials science

## Abstract

High electrical and thermal conductive metals (HETCM) play a key role in smart electronics, green energy, modern communications and healthcare, however, typical HETCM (e.g., Ag, Au, Cu) usually have relatively low mechanical strength, hindering further applications. Selective laser melting (SLM) is a potentially transformative manufacturing technology that is expected to address the issue. Ag is the metal with the highest thermal conductivity, which induces microscale grain refinement, but also leads to high internal stresses by SLM. Here, we select Ag7.5Cu alloy as an example to demonstrate that multi-scale (micro/meso/macro) synergies can take advantage of high thermal conductivity and internal stresses to effectively strengthen Ag alloy. The mimicry of metal-hardened structures (e.g., large-angle boundary) is extended to the mesoscale by controlling the laser energy density and laser scanning strategy to manipulate the macroscale internal stress intensity and mesoscale internal stress direction, respectively, to form mesoscale large-angle "grains", resulting in multiple mutual perpendicular shear bands during fracture. The presented approach achieved a significant enhancement of yield strength (+ 145%) and ductility (+ 28%) without post-treatment. The results not only break the strength-ductility trade-off of conventional SLM alloys, but also demonstrate a multi-scale synergistic enhancement strategy that exploits high thermal conductivity and internal stresses.

## Introduction

High electrical and thermal conductive metal (HETCM), such as, Ag, Au, Cu etc., has been widely used in key fields of medical science, smart electronics, modern communication (5G) and green energy (photovoltaic) due to its multi-functions in biomedical (antibacterial effect), electricity, thermology, optics and chemistry^1–5^. However, the expanding applications in high-tech fields of typical HETCM (such as Ag, Au) is hindered by its relatively low hardness, yield strength and high cost^6^.

As a common additive manufacturing (AM), selective laser melting (SLM) is well-known for its advantages in mechanical strengthening, precision manufacturing, and multi-scale precision control, which is expected to address the issue^7,8^. Many studies have reported that laser rapid solidification in SLM process facilitates grain refinement and thus improves metal strength^9–12^. The high thermal conductivity metal is expected to further increase the solidification rate, offering the possibility to improve the HETCM’s strength. Currently, multi-scale synergistic enhancement of mechanical properties of AM metals is a hot research topic in recent years^13–15^. The unique laser process used in metal AM results in complex hierarchical micro–macro structure^13,16^. Wang et al*.*^17^ have found that metallic materials produced by additive manufacturing experience complex stress and thermal gyrations along the build direction. This has the potential to produce complicated heterogeneous microstructures that may exhibit a wide variety of mechanical properties^17^. The results reveal a remarkable hierarchy of microstructures clarifies the relationships amongst different features and provides guidance for future structural manipulation of materials produced by additive manufacturing^17^. Wang et al*.*^13^ reported that austenitic 316L stainless steels via a SLM technique exhibit a combination of yield strength and ductility that surpasses that of conventional 316L steels. High strength is attributed to solidification-enabled cellular structures, low-angle grain boundaries, and dislocations formed during manufacturing, while high uniform elongation correlates to a steady and progressive work-hardening mechanism regulated by a hierarchically heterogeneous microstructure, with length scales spanning nearly six orders of magnitude. Zheng et al.^14^ confirmed that AM enables multi-scale integrated control of porous materials, from the nanometer to centimeter scale, for the manufacturing of porous hierarchical metamaterials^14^. Pham et al.^15^ reported the use of 3D printing to mimic certain micro-scale hardening mechanisms typically found in alloys and reinforced porous materials at a larger scale^18^. They deliberately mimicked the micro-scale structure (e.g., grain boundaries, precipitates and phases) into crystal-inspired lattices at the meso-scale to construct meso-scale lattice unit structures from nodes (analogous to atoms) and struts (analogous to atomic bonds) to improve the damage tolerance of porous materials^15,18^.

**Figure 1:**
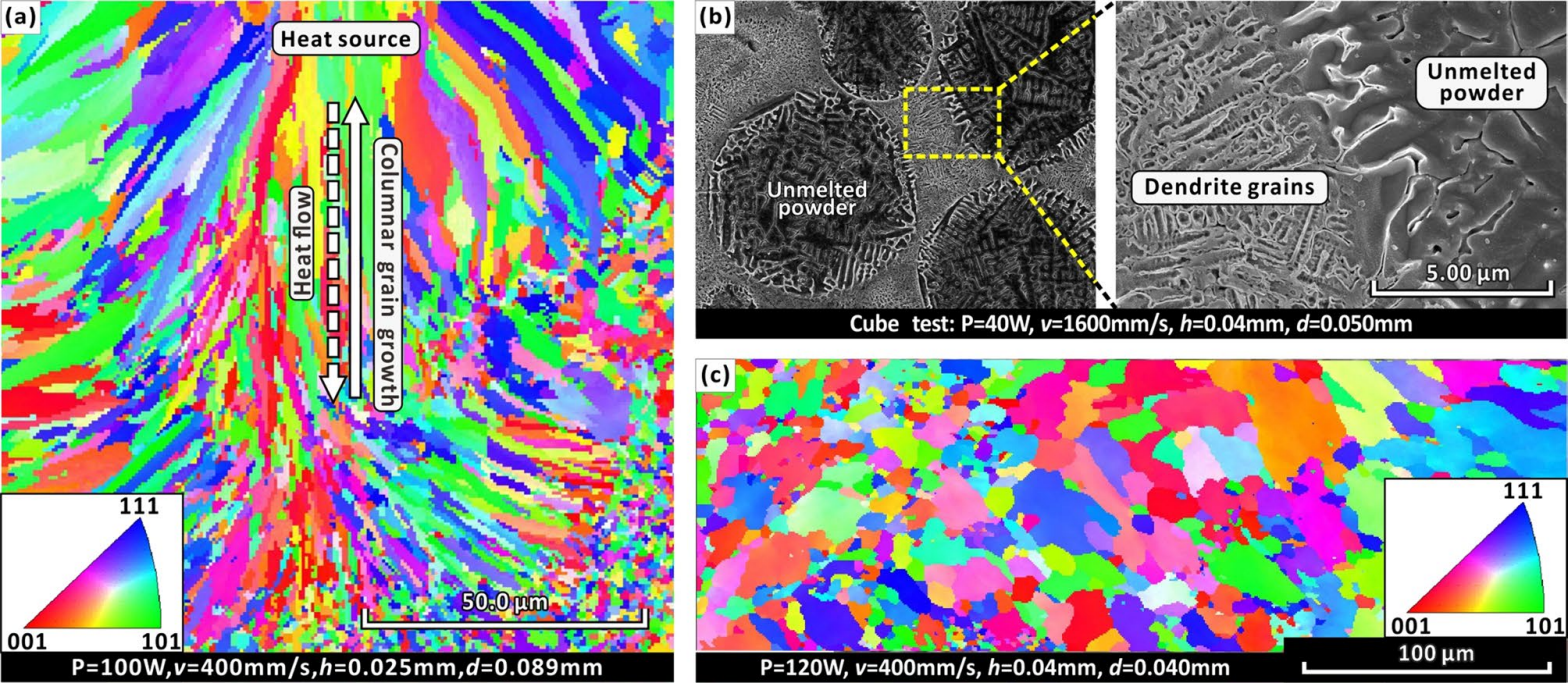
High* Q* Ag7.5Cu alloy forms a variety of microstructures under different AM parameters **(**see Fig. S2, Supplementary Information for details): (**a**) An EBSD-IPF map of directional columnar grains. The major axis of the grain is directed to the laser heat source. (**b**) A SEM image of dendrites in unmelted spherical powders and molten regions. (**c**) An EBSD-IPF map of equiaxed grain.

The SLM process control of high thermal conductivity metallic materials is challenging and is not conducive to mechanical enhancements at different scales^19,20^. The SLM manufacturability of silver alloys has been studied by related researchers^21^. Xiong et al*.*^21^ selected a 1 μm wavelength laser device and spherical Ag alloy powders. They have demonstrated it can overcome the challenges of high reflectivity and thermal conductivity in producing Ag parts at the various process parameters. They have achieved SLM processed Ag alloy to have a density as high as 96.7%. The SLM process leads to grain refinement and residual stress increase of the Ag alloy, which in turn, triple the Vickers hardness of the Ag alloy component when compared to the Vickers hardness obtained from the casting process^21^. Meanwhile, related research^22,23^ has shown that suitable wavelength lasers can increase the laser energy absorption of metal powders, opening up the possibility of improving the manufacturability of highly reflective metals (e.g., Ag). Several papers^24–27^ have been published on the effect of different emission modes (such as CW and PW emissions) on SLM processing. Caprio et al*.*^24^ confirm that CW emission provides a larger and more stable molten pool during the process. while PW emission might be problematic for porosity formation. However, like many high thermal conductive metals, silver alloy powder is exposed to a series of processing steps, particularly layer-by-layer rapid melting and solidification during SLM, which leads to inevitable high thermal gradients and internal stresses and associated defects^28–31^. On the one hand, the high thermal gradient will reduce the size of the constitutionally supercooled zone, which increases the difficulty in forming ultra-fine equiaxed grains^28,30,31^. On the other hand, it tends to induce intolerable columnar grains with long channels containing residual liquid^29,32,33^. Localized contraction of columnar grain can then cause pores to form thermal tears, thereby increasing stress cracking and reducing strength^29,31,34^. Therefore, grain refinement due to rapid solidification often increases the strength but reduces ductility through SLM^35^. Many approaches towards high strength often result in poor ductility, a dilemma known as the strength–ductility trade-off^8,13,35,36^. A variety of microstructures (Fig. 1, S2, Supplementary Information), including columnar grains (Fig. 1a), dendrites (Fig. 1b) and equiaxed grains (Fig. 1c) have been observed in the pre-study of SLM silver in this study, demonstrating the complexity of micro-scale control. Meanwhile, at the macro-scale, the localization of laser melting at high thermal conductivity and the directionality of the laser scanning track causes the formation of high internal stresses. The laser scanning track planning is also called laser scanning strategy, which is a unique molding method in metal AM. In this technique, the laser spot scans the metal powder to melt a dense line, these line scans accumulate to melt a dense planes, and the plane layers accumulate to melt a dense solid^21^. A unidirectional scanning strategy can cause internal stress defects induced by the concentration of stress directions^19,20^.

**Figure 2:**
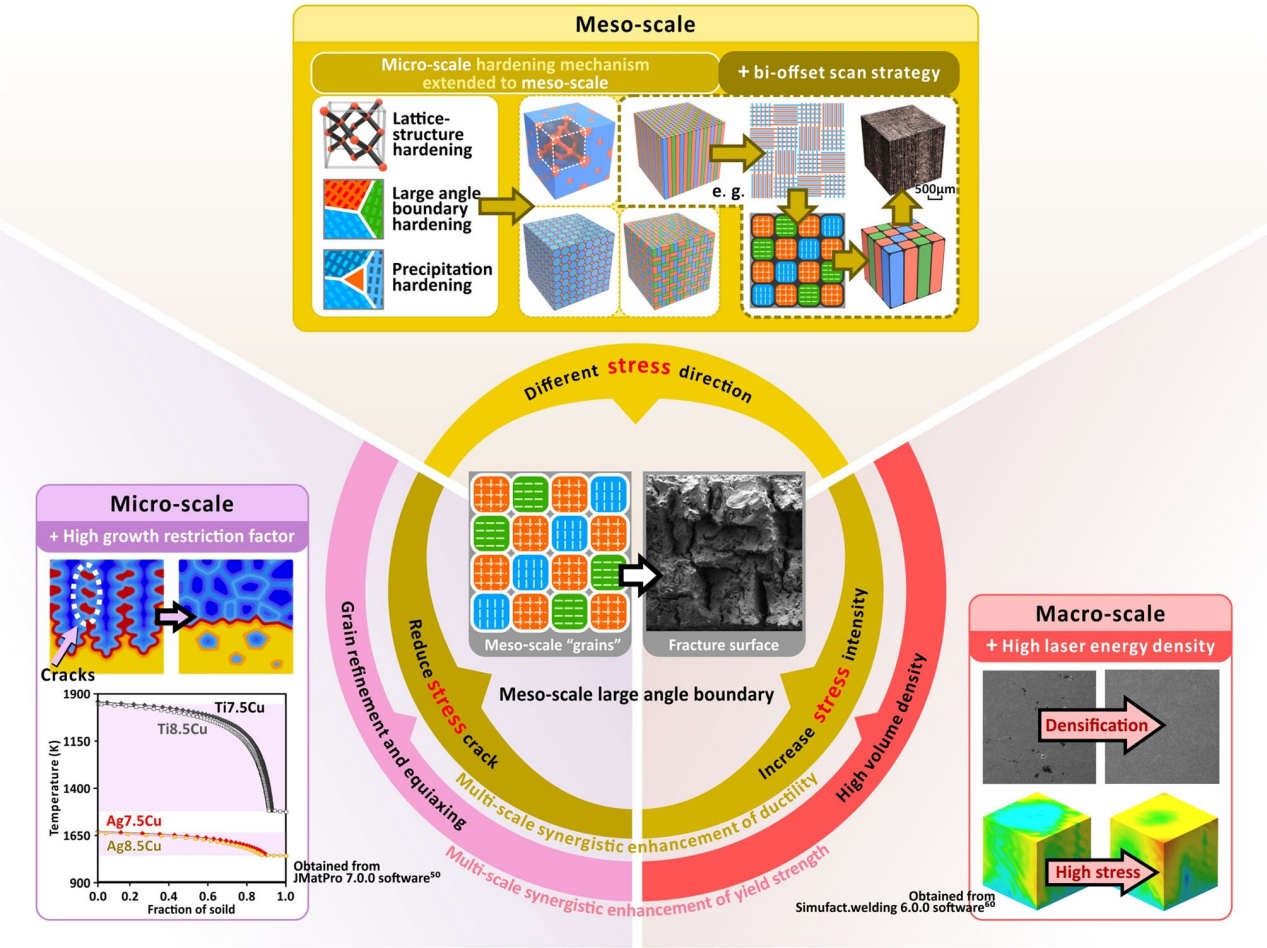
Schematic diagram of multi-scale synergistic reinforcement mechanism.

Here, we select sterling-silver alloy as an example to demonstrate that multi-scale (micro/meso/macro) synergies can take advantage of high thermal conductivity of Ag and high internal stresses by SLM to simultaneously improve the strength and ductility (Fig. 2). Most of the previous studies of mechanical reinforcement by deliberately manipulate multi-scale by AM have focused on porous materials^14,15^, whereas our research focuses on solid alloys. Similarly to the mimicry of structures within biological systems (or organic materials) in order to design bio-inspired materials^15,37–39^, in this approach, mimicking of the micro-scale metal hardening structure (e.g. large-angle boundary) is extended to the meso-scale, forming meso-scale “grains” with desired properties. Based on the high thermal conductivity of silver and the rapid solidification of laser leading to microscale grain refinement, the macroscale internal stress intensity and mesoscale internal stress direction in different domains (analogous to high-angle boundaries) are manipulated by controlling the laser energy density (LED) and designing laser scanning strategies, respectively, to form large-angle mesoscale "grains" with desirable properties, which promotes the formation of multiple shear bands perpendicular to each other at fracture, resulting in high-performance AM alloy parts (high strength, density and ductility).

## Results and discussion

### Effect of the composition of SLM Ag–Cu at the microscale

#### Effect of Ag alloying on microstructure

**Figure 3:**
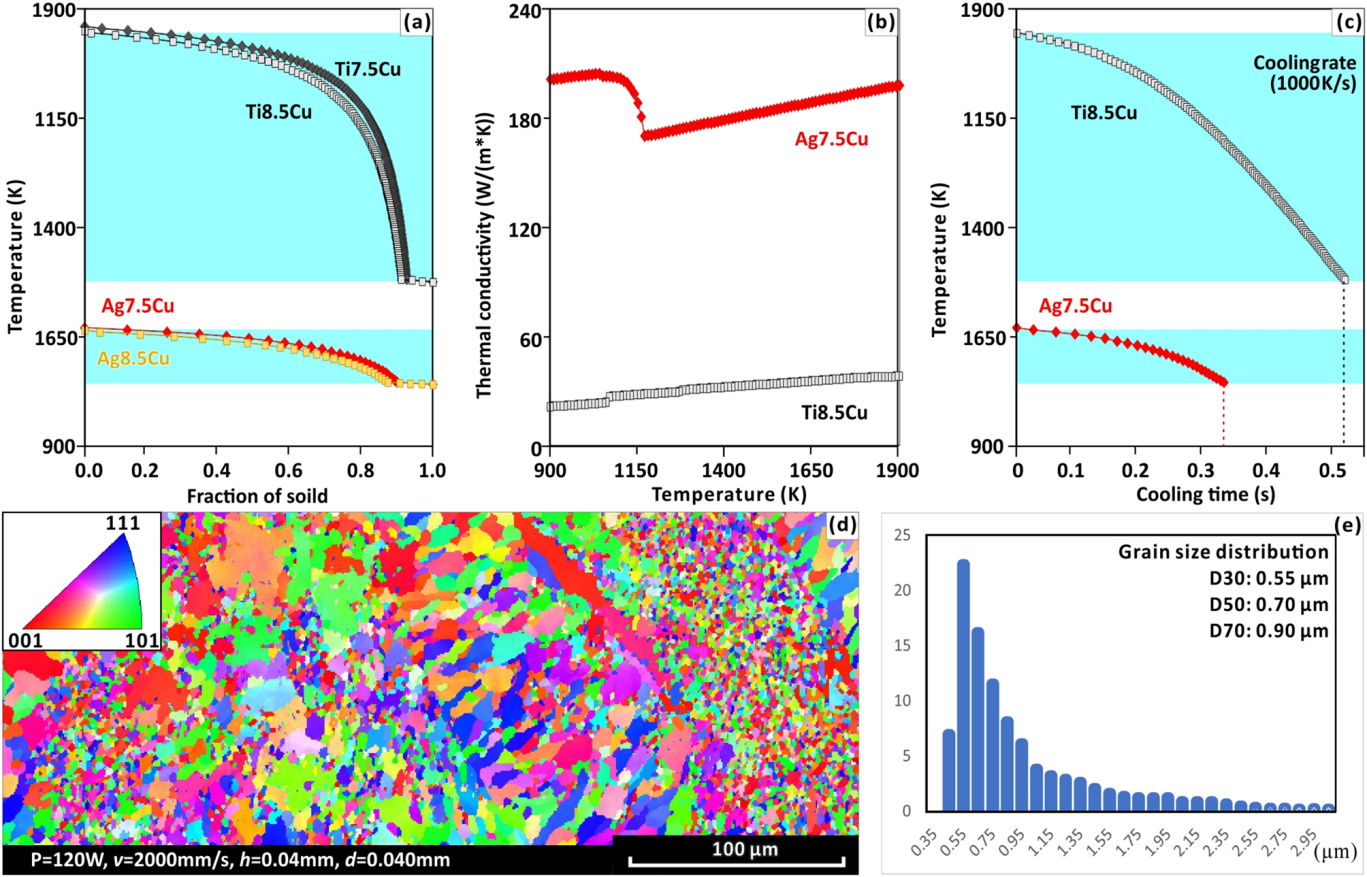
(**a**) Scheil-Gulliver solidification curves of Ti8.5Cu, Ti7.5Cu, Ag8.5Cu and Ag7.5Cu. (**b**) Thermal conductivity of Ti8.5Cu and Ag7.5Cu during solidification. (**c**) Cooling curves of Ti8.5Cu and Ag7.5Cu during solidification. All the three figures (**a**–**c**) are obtained from JMatPro 7.0.0 software^54^. (**d**) An EBSD-IPF map of ultrafine equiaxed grains. (**e**) Grain size distribution based on the EBSD-IPF map.

At the micro-scale, grain refinement, grain equiaxed and solid solution strengthening can be achieved by selecting a suitable solute of silver alloy. Research on using alloys to improve silver’s properties has a long history^6,40^. A silver alloy containing 7.5 wt.% non-silver solute is specified as the standard for sterling-silver, which is widely used as a conductor of heat and electricity, and standard coins and high-end crafts^41^. Ag and Cu have the same atomic packing properties and, similar lattice constants (Ag: 0.40 nm, Cu: 0.36 nm), providing an appropriate concentration of low-energy barrier heterogeneous nucleation sites ahead of the solidification front^34^. The rapid solidification by SLM lead to a significant non-equilibrium solute-trapping effect in supersaturated Ag alloy, which relieves the solubility limitations and facilitates solid solution strengthening in supersaturation^20,21,42^. During the solidification of alloys, segregation of solutes facilitates the formation of a constitutional undercooling zone ahead of the solid/liquid interface. “Secondary” nucleation within the constitutional undercooling zone can restrict the growth of the “primary” grains, resulting in grain refinement^29,30^. The grain-refining efficiency of solutes can be quantitatively expressed by its *Q* value^30,31,43^. Through the simulation of a Scheil-Gulliver solidification curve (Fig. 3a), Ag7.5Cu alloy is found to have a high Q value (32 K). According to interdependence theory^43^, Ag alloy with high Q value promote grain refinement and equiaxed. The grain-refining efficiency of AM Ag7.5Cu alloys stems from the capability of the Cu solute to establish a sufficiently large constitutional supercooling zone in front of the solid–liquid interface when the solute copper segregates^44^. Forming equiaxed grains can help reduce stress cracking and improve its suitability for metal AM^44^. Refined and equiaxed grains lead to short channels of fluid that can easily be backfilled compared to columnar grains, thereby reducing stress cracking and increasing strength^29,31,34^.

#### Effect of high thermal conductivity of Ag on microstructure

The high thermal conductivity of silver alloy and its small freezing range further promotes grain refinement. The laser process offers extremely high solidification rates compared to the casting process. Experiments have shown that the average grain diameter of cast silver alloys is 166.18 μm for the same alloy composition (Fig. S2a), while laser silver alloys can achieve an average grain diameter of as little as 0.70 μm (Fig. 3d-e). Generally, larger *Q* values mean a higher grain-refining efficiency^30^. According to the interdependence model (Eq. 1), in addition to high* Q* value, high solidification rates (*v*) also play an important role in grain refinement^43^.

1 $${d}_{gs}=\frac{D\cdot z\cdot \Delta {T}_{n}}{v\cdot Q}+\frac{4.6\cdot D}{v}\cdot \left(\frac{{C}_{l}^{*}-{C}_{0}}{{C}_{l}^{*}\cdot \left(1-k\right)}\right)+{x}_{sd}$$

where ${d}_{gs}$ is the grain size and $v$ is the solidification rate. The high thermal conductivity of silver increases the solidification rate ($v$). The high solidification rate ($v$) increases and the grain size decreases through a reductions in the first two terms of Eq. (1)^28^. According to physical property simulation, during the solidification process, Ag7.5Cu has six-fold greater thermal conductivity than that of Ti8.5Cu (Fig. 3b), and 78% narrower freezing range between its liquidus and solidus temperatures (Fig. 3b). Even at the same cooling rate, the solidification time of Ag7.5Cu is two-thirds of that of Ti8.5Cu (Fig. 3c). Therefore, in high* Q* alloy systems, the high thermal conductivity of Ag and narrow freezing range can induce further grain refinement and ultra-fine grains. In addition, the Scheil–Gulliver model can be used to determine the solidification path and freezing range and is often used to predict the likelihood of cracking during solidification^44^. Reducing the temperatures difference between the solidus and liquidus (freezing range) will also improve resistance to high internal stress tearing^34^. Zhang et al.^44^ showed that ultrafine equiaxed grains (9.6 μm) can be produced in Ti8.5Cu alloys (high *Q* value, without nucleants) and successfully realized high-strength titanium alloys^45^. Although, the* Q* value of Ag7.5Cu (32 K) is lower than that of Ti8.5Cu (62 K), Ag7.5Cu has a higher thermal conductivity and forms finer equiaxed grains (0.70 μm, Fig. 3d) at (LED) of 37.5 J/mm^3^, which is 1/14 as that of Ti8.5Cu (9.6 μm) reported by Zhang et al.^44^, regarded as the finest grain in existing SLM literature^46–53^.

### Effect of LED on internal stress intensity at the macroscale

**Figure 4:**
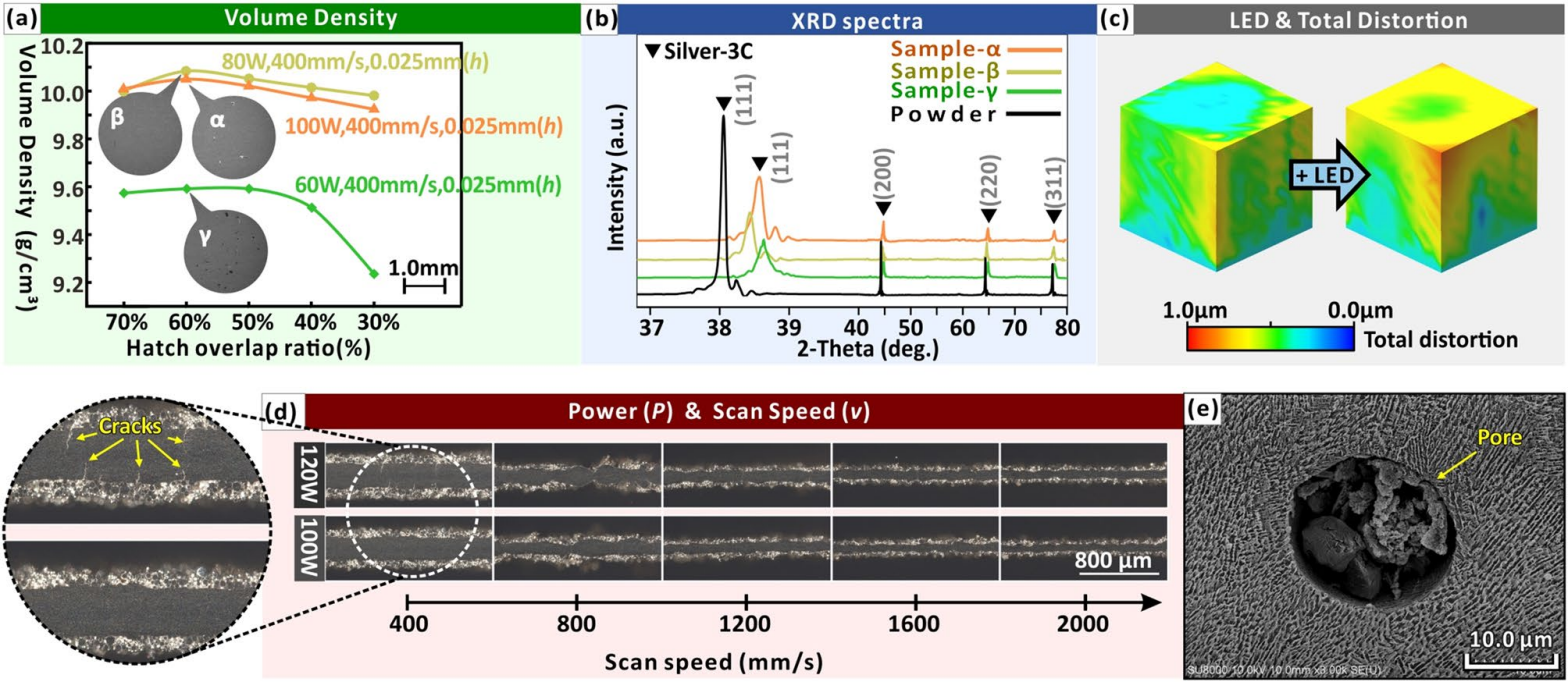
Effect of process parameters on morphology, volume density, distortion and defects (See Fig. S3, Supplementary Information for details). (**a**) Effect of process parameters on volume density. In general, LED is proportional to the volume density. According to LED (Eq. 1), LED is directly proportional to the laser power, and inversely proportional to scan speed, layer thickness and hatch distance. (**b**) XRD spectra of Ag alloy powders and representative sample sample-α, -β, -γ. (**c**) Effect of LED on total distortion by FEA (the figure is obtained from Simufact.welding 6.0.0 software^64^). An OM image of excessive LED results in the formation of (**d**) cracks and (**e**) a SEM image of micro-pores.

To achieve multi-scale synergistic strengthening, the effect of LED on macroscale internal stress is analyzed. At the macro-scale, LED affects not only the volume density, but also the internal stress intensity of the entire AM component (optimization process of high-volume density process parameters; refer to Methods and, Fig. S3, Supplementary Information). Generally, LED is proportional to the volume density and is a basic parameter setting^8,21,42,55–59^. Through the cube test, two groups of parameters that produced the high-volume densities are selected as optimum, while one group of parameters that produced a lower volume density is used as reference. These three groups of parameters are mentioned repeatedly, so the samples prepared are named α, β, and γ, respectively (Fig. 4, 7, 9, 10; Detailed parameters for the three samples are given in the supplementary information, Fig. S3f.). In this study, sample-β had the highest volume density (10.08 g/cm^3^; Fig. 4a), but it is not the sample with the highest LED. The effect of parameters on the forming performance is not merely a summary of the LED. Excessive LED causes stress cracks and pores, thereby reducing volume density and mechanical strength (Fig. 4d-e). Similar to metal welding process induces melting and conducts, local expansion and shrinkage during laser AM result in deformation and internal stresses^60^. High internal stresses in AM processes lead to stress cracking when the LED is extremely high (120 W, 400 mm/s; Fig. 4d). At less than maximum volume density, a higher LED helps to reduce internal stresses, since a reduced thermal expansion mismatch between solidified melt track and surrounding material^61^. However, when the volume density reaches its maximum, a further increase in the LED results in increased internal stresses between melt pools of local expansion. The same direction of the laser scanning track during the forming process leads to the accumulation of internal stresses in the same direction and increases the macroscopic internal stress intensity. According to finite element analysis (FEA) of AM process simulation, a higher LED also leads to higher deformations of fully dense alloys due to internal stresses (Fig. 4c). The XRD patterns of samples-α, -β, -γ shift towards the higher diffraction angles compared with that of Ag alloy powder, also indicating higher internal stresses^21,62,63^. The internal stress acts on the grain boundaries, which may influence the lattice parameters, causing the 2θ angles shift to high values^63^.Apart from the unprocessed silver powder, sample-β (with the highest volume density) had the smallest 2θ angles. Therefore, sample with the highest volume density (sample-β) tended to develop relatively low internal stresses, while further increases (sample-α) or decreases (sample-γ) in the LED all increased internal stress.

### Effect of laser scanning strategy on internal stress direction at the meso-scale

**Figure 5:**
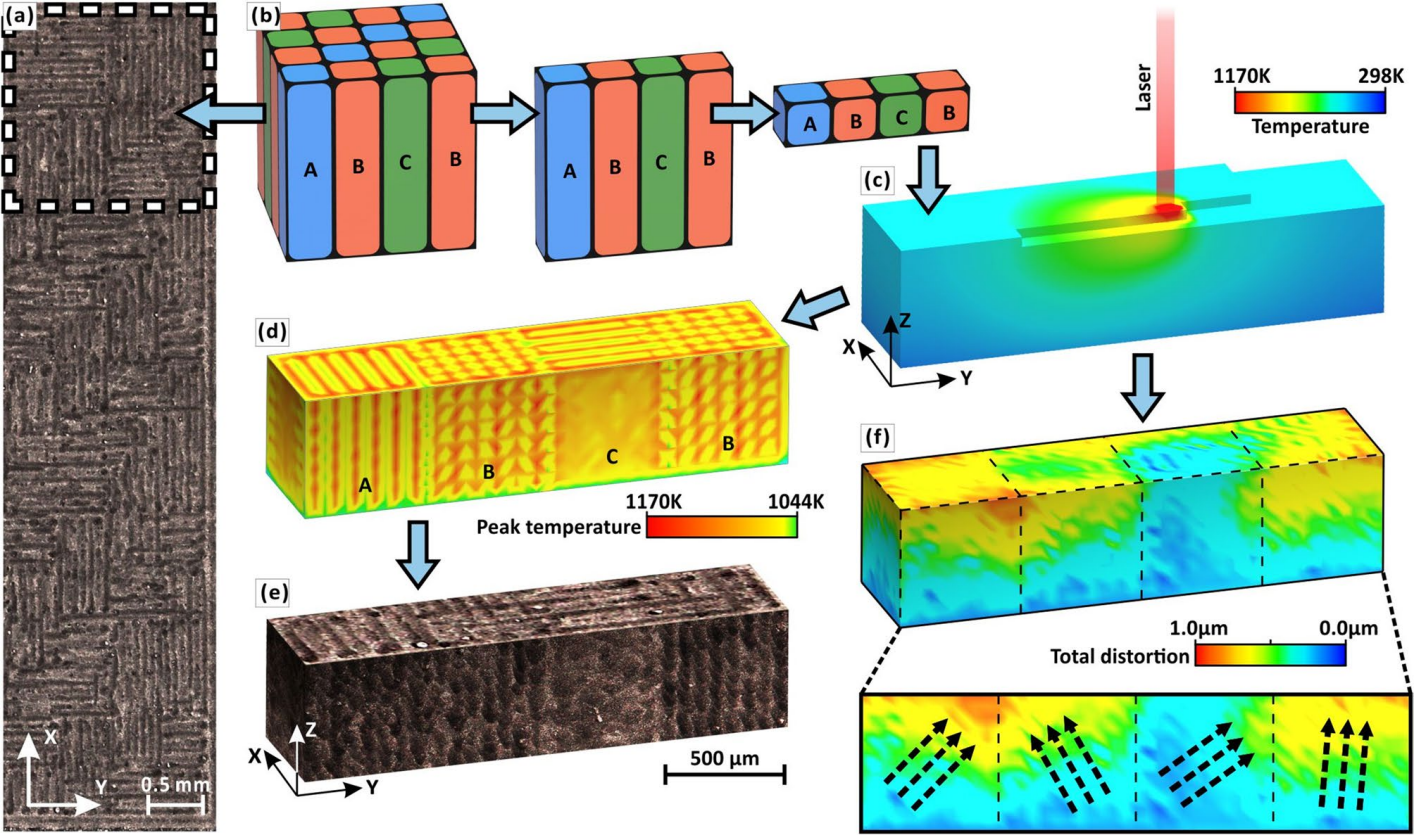
(**a**) OM images of single-layer morphology. (**b**) 3D view of meso-scale columnar grains induced by three scan types of small domains: X-axis progressive scan (type A), Y-axis progressive scan (type B) and cross-scan (type C). (**c**) Thermal field simulation in forming process. FEA of peak temperature distribution (**d**) and total distortion (**f**) by bi-offset scanning strategy. (**e**) Morphology of sample-α observed by OM corresponding to the peak temperature distribution by FEA. (**c**), (**d**) and (**f**) are obtained from Simufact.welding 6.0.0 software^64^.

The mimicry of metal-hardened structures (e.g., large-angle boundary) is extended to the mesoscale by designing a bi-offset scanning strategy to manipulate the differences in internal stress directions of different domains, to form "grains" with large-angle boundaries at the meso-scale, so the meso-scale "grains" also refer to the domains scanned by the same type. Notably, past research on large-angle boundary effects has focused on the micro-scale. A single grain consists of a unit cell of the same type and orientation, but most alloys consist of multiple grains, each of which contains different lattice orientations with its neighbors. The orientation of neighboring lattices is important in forming a large-angle boundary, which prevents the propagation of cracks and strengthens the alloy^15^. Related literature^18^ also indicates that mechanical property differences between grains can enhance large-angle boundary effects. Pham et al*.*^15^ use the hardening mechanisms found in crystalline materials to develop architected materials that are robust and damage-tolerant, by mimicking the micro-scale structure of crystalline materials—such as grain boundaries. Here we achieved different directional internal stresses (Fig. 5) between adjoining mesoscale domains by creating a boundary, similar to the boundary between the two adjoining micro-scale grains. The laser scanning strategy is a unique molding method in metal AM. The design method of a bi-offset scanning strategy is detailed in the Methods. Figure 5a shows the single layer morphology after metallographic polishing and etching. At the same time, FEA of the AM process according to the bi-offset scanning strategy clearly shows the peak temperature distributions of the three scanning types (A, B, C), which are in lined with the OM image (Fig. 5d-e). Different metallographic morphology of aforementioned types (same LED) reflects variations in mechanical properties among domains (analogous to different “grains”; Fig. 5e)^21,62,63^. It is also observed that the direction of deformation at the boundary between mesoscale “grains” changes significantly (Fig. 5f) which stands for the direction differences of internal stress concentrations. Therefore, by designing bi-offset scanning strategy, the difference in internal stress direction of different domains is successfully achieved, extending the mimicry of metal-hardened structures (e.g., large-angle boundaries) to the meso-scale.

### Multi-scale synergistic mechanical enhancement

**Figure 6:**
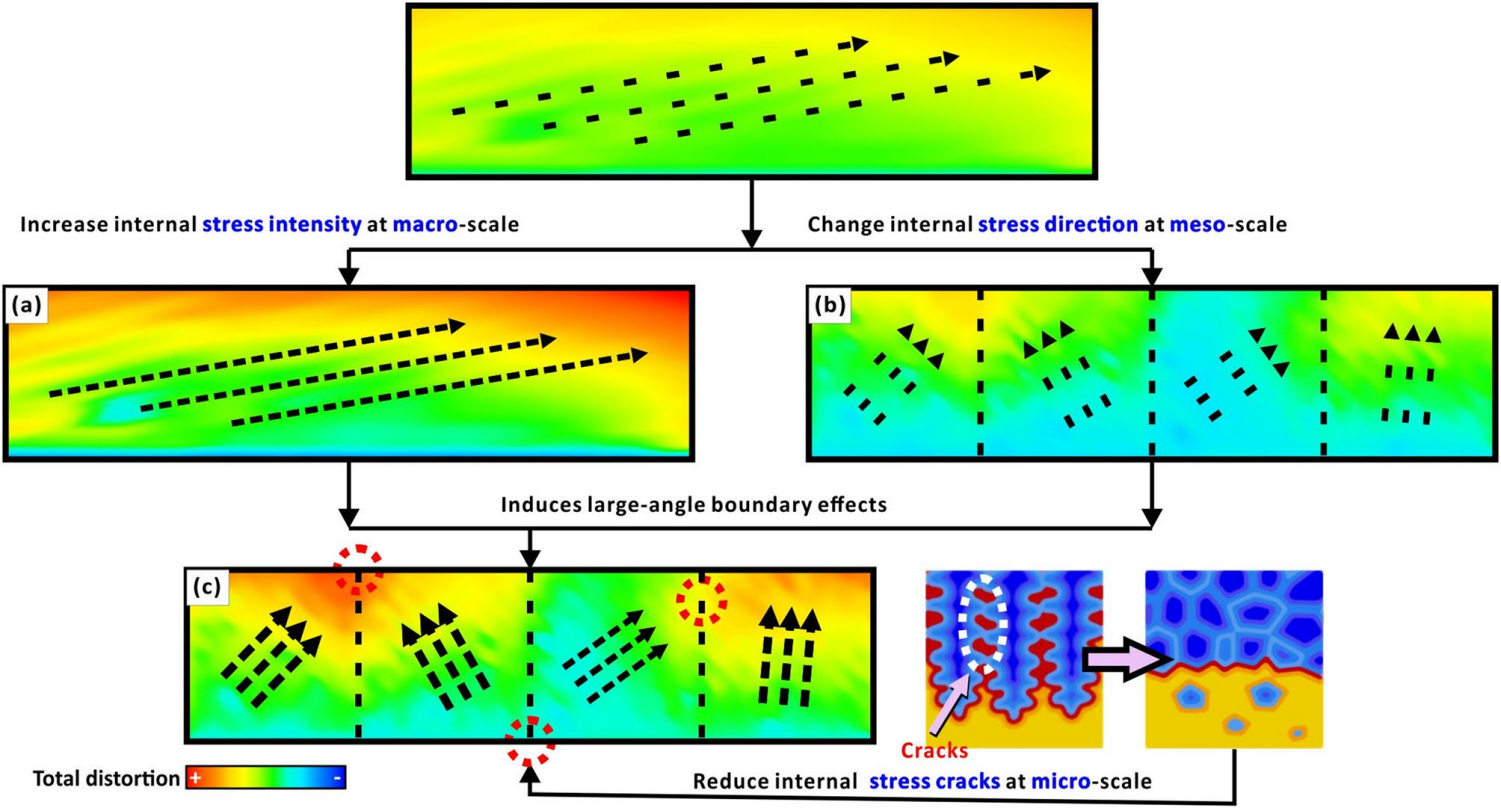
Schematic diagram of multi-scale (macro: (**a**), meso: (**b**), micro: (**c**)) synergistic reinforcement mechanism. All figures are obtained from Simufact.welding 6.0.0 software^64^.

The formation of multi-scale synergistic enhancement effects, at the macroscale, is due to the increase in LED increasing internal stress intensity (Fig. 6a). At the meso-scale, due to the bi-offset scanning strategy, mechanical properties (especially internal stress direction) in each domain are different from each other (Fig. 6b). Although the macro- and meso-scale synergies increase the large-angle boundary effect, they also increase the tendency for internal stress cracking. Therefore, at the microscale, grain refinement and equiaxing are indispensable in preventing stress induced defects caused by the introduction of high internal stresses during the manufacturing process (Fig. 6c)^29,31,34^. If an alloy retains a large volume of cracks caused by internal stresses it will have a detrimental effect on its strength.

**Figure 7:**
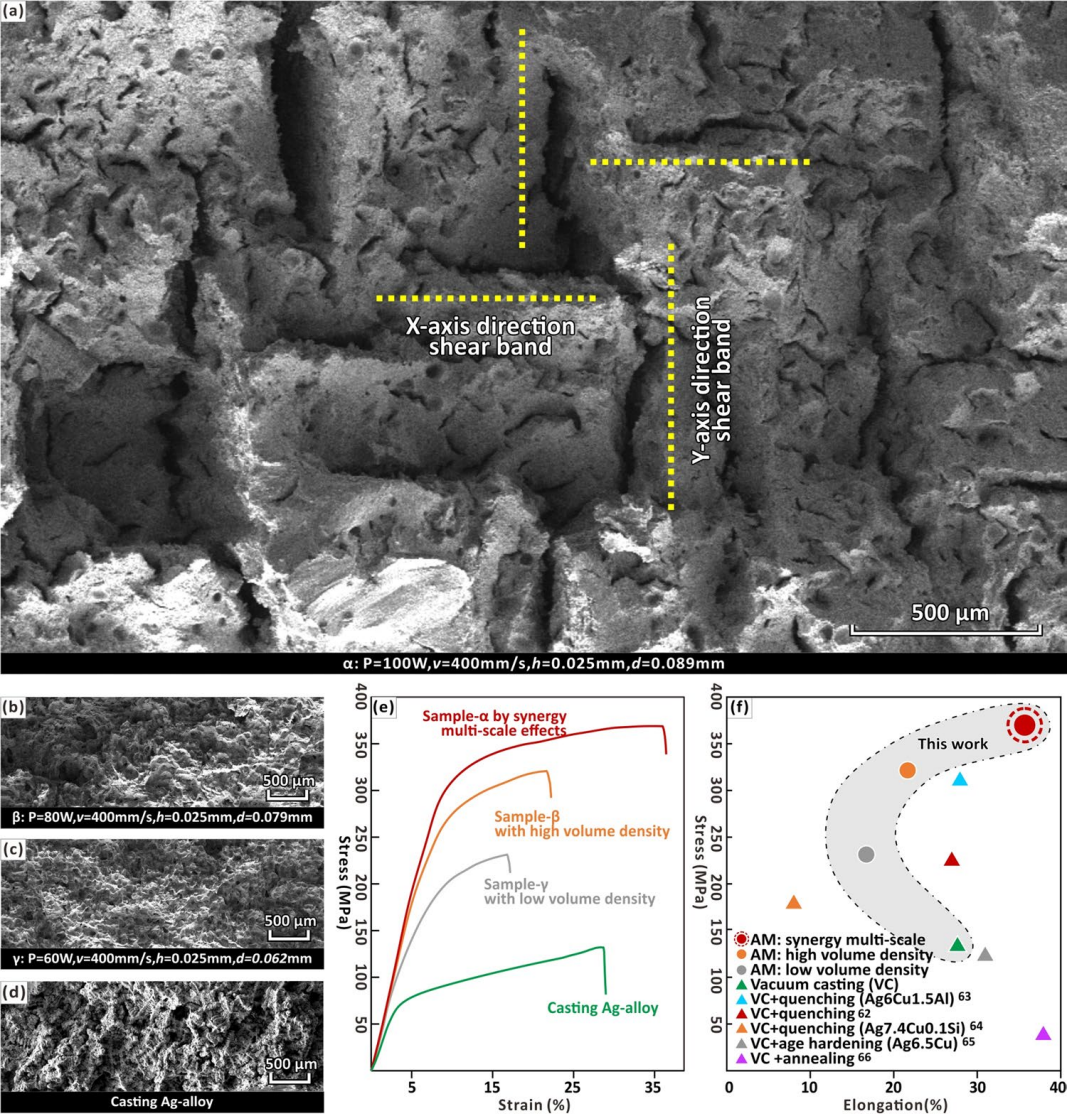
SEM image of fracture surface morphology of (**a**) sample-α with synergy multi-scale effects, (**b**) sample-β with high volume density and (**c**) sample-γ with low volume density and (**d**) casting sample. (**e**) Stress–Strain diagrams of representative samples produced by metal AM and casting. (**f**) Summary of the elongation versus stress for silver alloy (examples of unspecified components are Ag7.5Cu alloys)^66–70^.

The fracture surface of sample-α had an obvious perpendicular shear band in the SEM image (Fig. 7a). The morphology of the shear band is consistent with the mesoscale “grain” boundaries mentioned above, caused by different scanning types. Cracks did stop at incoherent large-angle boundaries, preventing fast brittle fracture by impeding the dislocations move across the “grain”^15,65^. It was also observed that the fracture surface morphology of sample-β (Fig. 7b), -γ (Fig. 7c) and casting (Fig. 7d) do not form mutually perpendicular shear bands and their tensile strength and ductility are lower than that of sample-α.

Using this multi-scale reinforcing strategy, sample-α with mesoscale “grains” revealed the highest yield strength and ductility during tensile testing in this work (Fig. 7e). Compared with cast counterparts, tensile strength increased by 145% and ductility increased by 28% (Fig. 7e; Table S2, Supplementary Information). Therefore, without the need of composition adjustment (e.g. adding silicon^66^ and aluminium^67^) and post-treatments (e.g. quenching^66^, age hardening^68^ and annealing^69^), the presented multi-scale synergistic enhancement method break the strength–ductility trade-off of metal AM parts^8,13,35,36^, achieve a significantly improvement than conventional method (Fig. 7f)^8,13,35,36^.

**Figure 8:**
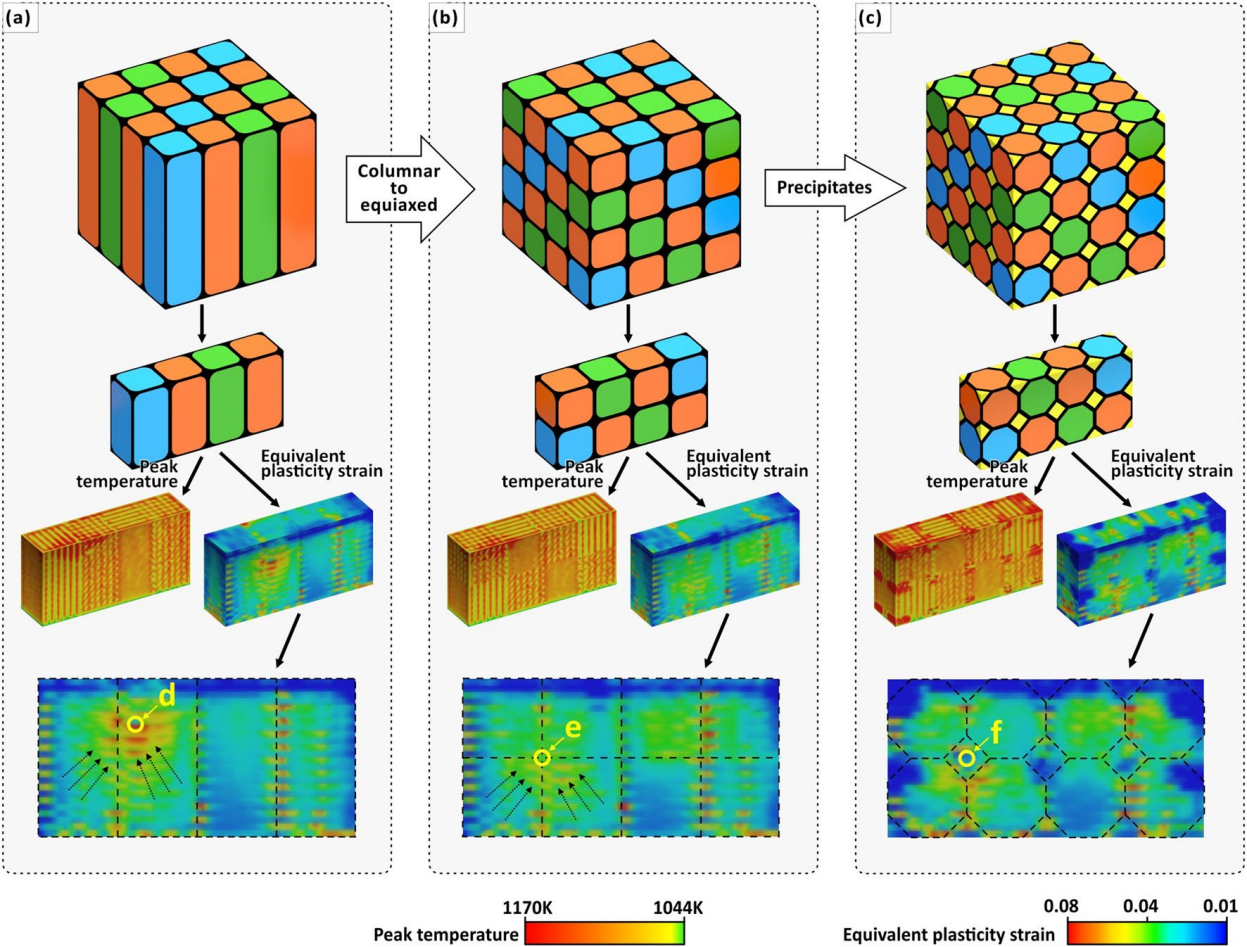
FEA of the peak temperature distribution and equivalent plastic strain distribution of (**a**) meso-scale columnar “grains”, (**b**) equiaxed “grains” and (**c**) equiaxed “grains” with precipitates introduced. (**d**) localized region of excessive stress emerged by residual stress accumulation in single direction. (**e**) localized region of high stress at the meso-scale “grain” boundary. (**f**) precipitate domains. All figures are obtained from Simufact.welding 6.0.0 software^64^.

As an extension of this method, tailoring the scanning strategy can produce not only the large angle boundary (Fig. 8a), but also other types of metal hardening structures (e.g. meso-scale equiaxed “grain” (Fig. 8b) and precipitation hardening (Fig. 8c)) inspired by micro-structure of crystalline materials. Pham et al.^15^ have shown that the designing meso-scale structure can strengthen materials by mimicking the micro-structure of crystalline materials—such as grain boundaries, precipitates and phases. The study^15^ suggests that these crystal-inspired meso-scale structures are as important for their mechanical properties as are crystallographic micro-structures in metallic alloys. In our study, FEA have shown the predictability of scanning strategy on its thermal and mechanical effects (Fig. 5d,e). On this basis, FEA can be used to further verify and optimize the meso-scale control strategy to achieve the desired “grain” structure and performance and reduce the number of precious metal AM experiments. The bi-offset scanning strategy designed in this experiment forms columnar “grains” along a < 001 > direction (Z-axis; Fig. 8a), which are more inclined to introduce anisotropy. Therefore, FEA is used to demonstrate the scanning strategy of further offset at equidistant layer thickness can achieve an equiaxed “grain” (Fig. 8b, Video S1, Supplementary Information). By comparing the plastic strain distribution of columnar and equiaxed “grains”, it is shown that the latter alleviates the residual stress usually occurs in single-direction scanning (Fig. 8: points d, e). Furthermore, we demonstrated the possibility to produce precipitation hardening by adjusting the laser parameters at “grain” boundaries (Fig. 8c). In precipitate-hardened alloys, precipitates act as obstacles to the movement of dislocations thereby enhancing mechanical properties^15^. Laser parameters (such as laser power and scanning speed) can control the mechanical properties (such as hardness) of AM silver alloy^21^. We introduced precipitation strengthening into a mesoscale “grain” structure by producing embedded “precipitate domains” using different laser parameters from equiaxed “grains” (Fig. 8c). In FEA, the thermal and mechanical characteristics of “precipitate domains” (Fig. 8c: point f) are significantly different from the adjacent “grains”. The introduction of precipitate domains creates dislocation obstacles, which are expected to prevent similar problems of a rapid reduction in stress induced by slip activity in single crystals^65^. For FEA simulations of the AM process related to different scanning strategies, see Video S1, Supplementary Information. However, although some reports suggested that these studies of crystal-inspired meso-scale structures are no less important than microstructures in mechanical strengthening^15^, we realize that the two scales cannot be simply equated and that future studies are needed to explain their strengthening mechanisms more fully.

**Figure 9:**
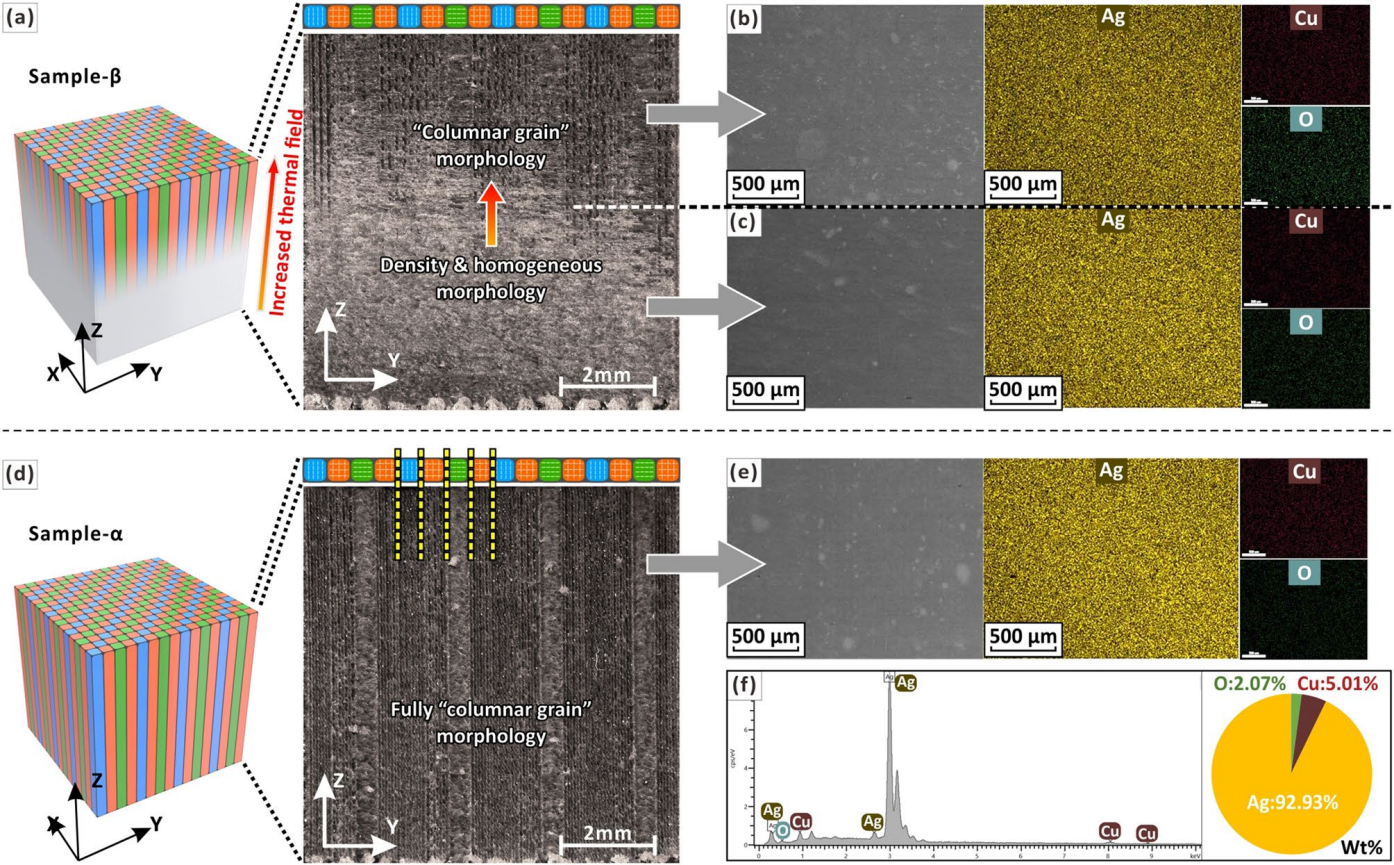
(**a**) An OM image showing morphology of sample-β with the highest density in the ZY-plane: continuous laser processing causes the thermal field to gradually increase in the Z-axis direction. (**b**) A SEM image showing “columnar grain morphology” and the corresponding EDS maps. (**c**) A SEM image showing “density & homogeneous morphology” and the corresponding EDS maps. (**d**) An OM image showing morphology of sample-α with a fully meso-scale “columnar grain”. (**e**) A SEM image showing “columnar grain morphology” and (**f**) the corresponding EDS analysis.

Further analysis shows that multi-scale synergistic enhancement of ductility requires the interaction of three length-scale effects (Fig. 6) and designing a bi-offset scanning strategy at the meso-scale only is not sufficient to form large-angle “grains” (Fig. 9a). By comparing samples α and β, the syngenetic strengthening mechanism can be better illustrated (Fig. 9a,b). The two samples have the same alloy composition, bi-offset scanning strategy and both with high volume density. However, their morphological and mechanical properties (Fig. 5, 7) are significantly different. In Fig. 9a,d, “density & homogeneous morphology” and “‘columnar grain’ morphology” are the melt pool morphologies formed by two different process parameters or thermal field. The "columnar grain" also refers to the domains scanned by the same type of island strips. By observing the region of “density & homogeneous morphology”, there is very little porosity in this region and the morphology tends to be the same in different regions. Whereas, the “‘columnar grain’ morphology”, are affected by the bi-offset scanning strategy and the morphology varies significantly from region to region, which is longitudinally banded and has a slight increase in porosity. According to the two sample parameters, two cubes are polished and etched in the ZY-plane. Sample-α has a full mesoscale “columnar grain” morphology matching the bi-offset scanning strategy (Fig. 9d), and it only break in the “columnar grain” area. This indicates that the “columnar grain” morphology region, which can induce multiple shear bands, results in a greater yield strength and ductility. While sample-β has a “columnar grain” morphology in the upper half, but a dense and homogeneous morphology in the lower half (Fig. 9a), where then become a weakness point in tensile test. The fracture surface is flat and homogeneous accounting for low ductility (Fig. 7b,e). The gradient change of sample-β in the Z-axis directions is mainly because the layer-by-layer fabrication leads to multiple thermal cycles, while the heat dissipation of the lower part is sever than the upper part^44^. The increase in the gradient of the thermal field causes sample-β’s morphology to change from dense and homogeneous to meso-scale columnar “grains” (Fig. 9a). The LED of sample-α is higher than that of sample-β, which is equivalent to providing a higher thermal field, hence a full “columnar grain” morphology is formed (Fig. 9d). Further increasing the LED (thermal field) in the highest volume density parameter range also promotes an increase in internal thermal stresses (Fig. 4b,c). Therefore, the formation of meso-scale “grains” is accompanied by an increase in internal stress intensity by increasing the LED at the macro-scale and a difference in internal stress direction by designing a bi-offset scanning strategy at the meso-scale (Fig. 5). At the microscale, combined with the high Q value, the small freezing range can help reduce thermally induced stress cracking resulted from high LED and different internal stress direction of different domains. Meanwhile, ultra-fine equiaxed grains (0.30 μm) and solid solution strengthening of Ag7.5Cu cause yield strength enhancement and substantial hardening during plastic deformation (Fig. 7e). This capacity to constrain deformation at the micro-scale and prevent the rapid propagation of slip minimizes the decreases in stress^71^ and strengthens the meso-scale “grain”. Finer equiaxed micro-structures allow easier grain rotation and deformation, providing a method to accommodate strain, thus preventing crack initiation and growth^34^. Consequently, yield strength and ductility are both affected to achieve a multi-scale synergistic enhancement effect (Fig. 2).

**Figure 10:**
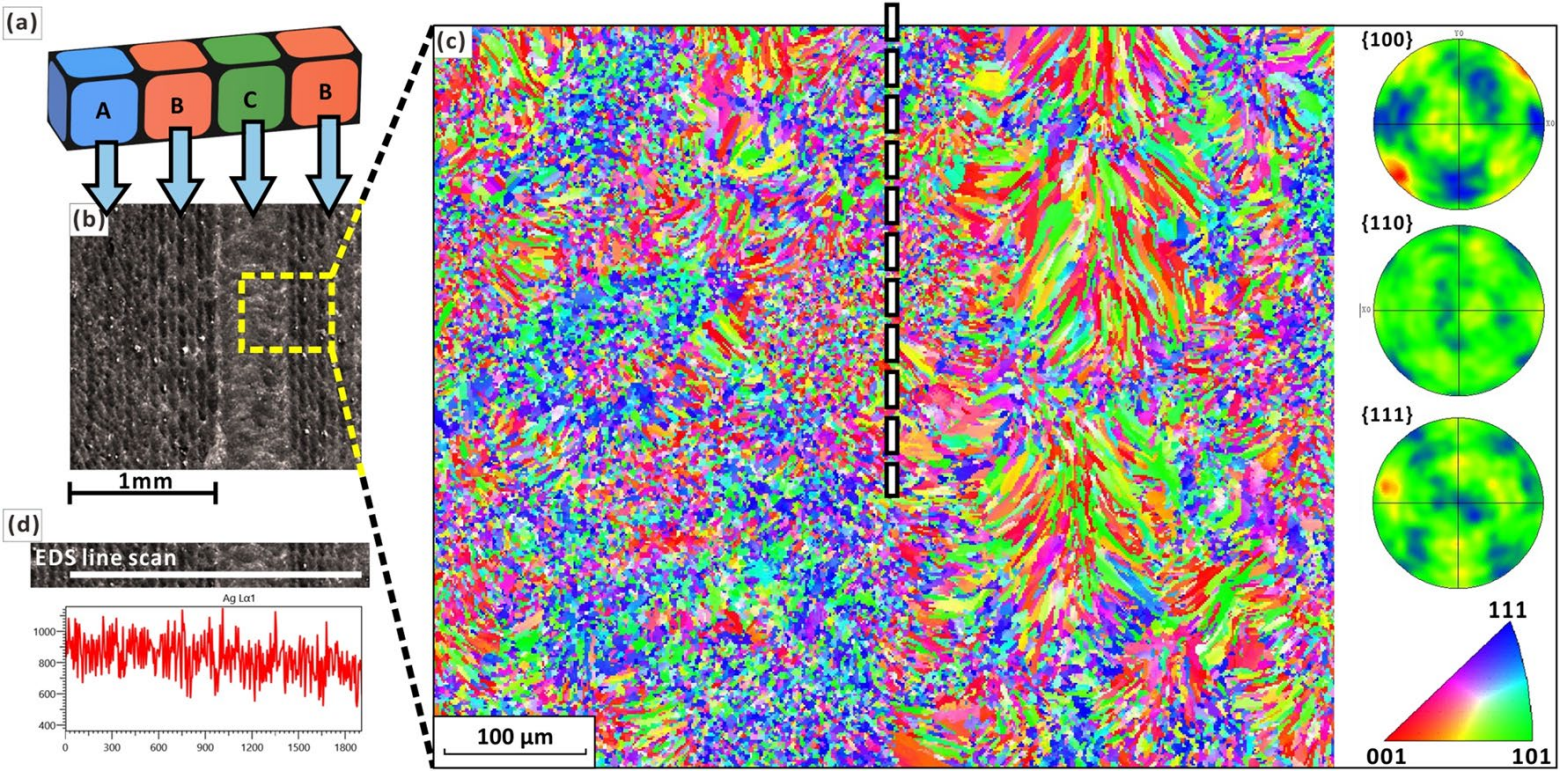
(**a**) 3D view of three scan types of small domains: X-axis progressive scan (type A), Y-axis progressive scan (type B) and cross-scan (type C). (**b**) Molten pool morphology of sample-α observed by OM. (**c**) EBSD mappings and (**d**) EDS line-scan of different scan types of small domains.

Furthermore, by observation of the OM images and EBSD-IPF maps (Fig. 9a,d; Fig. 10a–c), the heterogeneity of the melt pool morphology and grain structures are evident on the meso-scale scanned domain, which indicates that the setting of the laser parameters can control different domains with different types of grain structures, thus forming different types of domains at the meso-scale. For example, many columnar grains are observed in the scanned domain of type B while a large number of fine equiaxed grains are observed in the scanned domain of type C (Fig. 10b,c). There is a more distinct boundary between the scanned domains of type B and type C (Fig. 10b,c). Also, elements such as silver, copper and oxygen were detected by EDS. Of these, elemental oxygen may have been introduced into the as-built sample during the printing process (Fig. 9f). However, local chemical inhomogeneities at the meso-scale were not evident when elemental mapping was carried out (Fig. 9b,c,e). By EDS line scan, no significant compositional changes were observed at the outer boundaries of island strips (Fig. 10d). This suggests that the elemental distribution tends to be homogeneous on the mesoscale, although heterogeneity in melt pool morphology and grain structures can be observed on the meso-scale scanned domain (Fig. 10b,c). This may be due to four reasons. (1) The extremely high solidification rate within the very small local laser melt pool is not conducive to the separation of the different elemental components due to the extremely high thermal conductivity of silver. Several studies^6,72^ have shown that high cooling rates can be used to effectively prevent segregation and increase the solubility of the alloying elements, resulting in supersaturated silver alloys with high strength. (2) The small melt pool formed by partial melting prevents extensive composition segregation. (3) The study material, Ag7.5Cu, is only a binary alloy with a relatively simple composition. Therefore, the possibility of many different elements segregating in different regions is lacking. (4) Trace elements may not be readily detectable.

## Conclusions

We proposed multi-scale synergistic reinforcement strategies to make good use of high thermal conductivity of silver and internal stress during SLM processes to simultaneously improve the strength and ductility, thus breaking the strength-ductility trade-off of conventional SLM alloys. The study on nature and the formation mechanisms of the meso-scale structural heterogeneity, such as heterogeneity of melt pool morphology, are also explored. Through controlling the internal stress intensity at the macro-scale, internal stress direction of different domains at the meso-scale, mimicking of the metal hardening structure (e.g., large-angle boundary) is extended to the meso-scale, forming meso-scale “grains” with desired properties. Without the need to post-treatments, the presented approach revealed the highest yield-strength (+ 145%) and ductility (+ 28%) compared to that of casting. Meanwhile, the high thermal conductivity of Ag and high growth restriction factor (Q) solute of Cu are induced to further refine the grains, thus strengthening the meso-scale “grains”. By the synergistic multi-scale control, the silver alloy forms multiple shear bands perpendicular to each other during fracture, which effectively improves the mechanical properties and achieves higher yield strength (+ 145%) and higher ductility (+ 28%) than conventional silver alloy castings. In this case, Au7.5Cu parts achieved significant mechanical enhancement without complex AM equipment or post-processing. Besides, the FEA also demonstrate the feasibility to control the mechanical properties by mimicking various micro hardening mechanisms and designing different “grain” structures, even bionic structures. The proposed multi-scale synergistic reinforcement method described here can also be extended to other alloys with high thermal conductivity requiring high mechanical performances.

## Methods

### Powder materials

**Figure 11:**
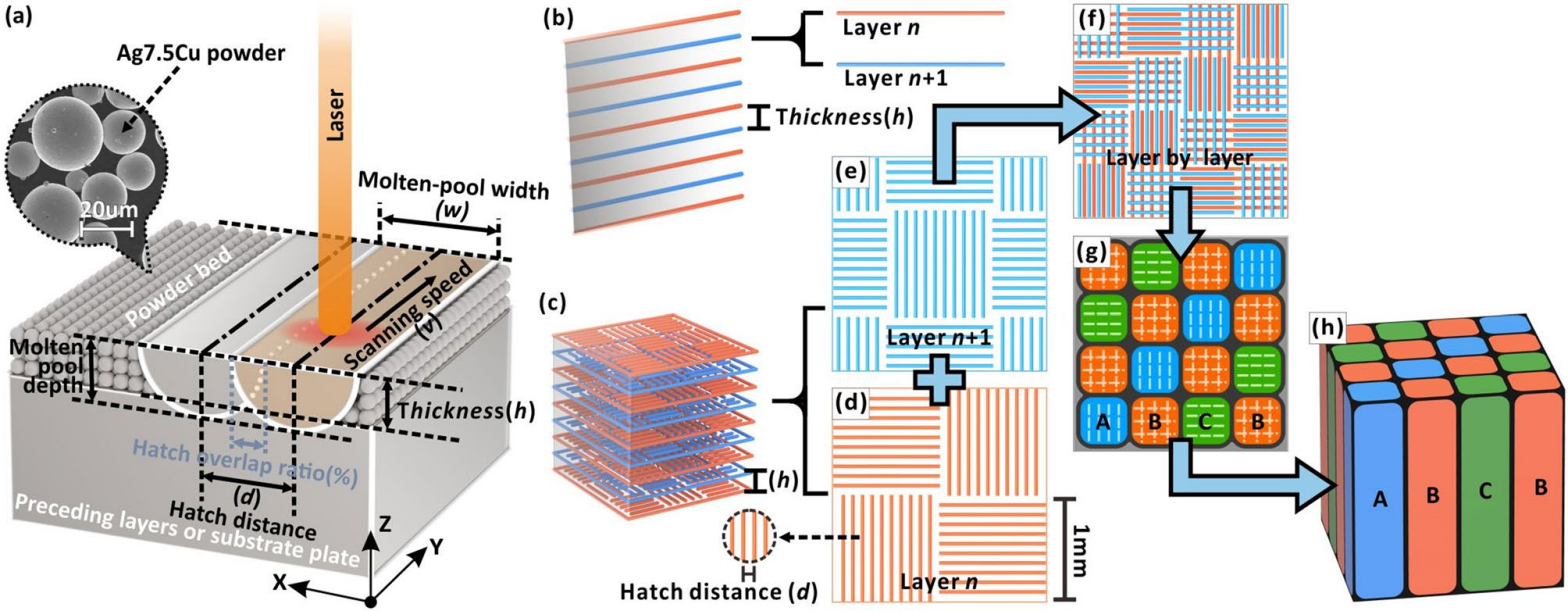
Schematic of (**a**) process parameters, (**b**) single-wall and (**c**) cube tests. (**d**–**h**) Schematic of bi-offset scanning strategy. (**d**) Angular-offset scanning in the XY-plane: The directions of progressive scanning are perpendicular to each other between adjacent square regions with 1.0 mm sides. (**e**) Position-offset scanning on the next layer: After being offset by $\sqrt{2}$/2 mm in the 45° direction on the XY plane, the scanning is performed in the same manner as the previous layer. (**f**) Three scan types form smaller square domains with 0.5 mm sides after layer-by-layer deposition. (**g**) Schematic of three scan types of small domains: X-axis progressive scan (type A), Y-axis progressive scan (type B) and cross-scan (type C). (**h**) 3D view of meso-scale columnar “grains” induced by the scanning strategy.

Ag7.5Cu alloy powder prepared by the gas atomization method was purchased from the Legor Group. (Table 1; Fig. 11a, S1, Supplementary Information). 92.5 wt.% silver alloy, also known as sterling-silver, is the standard for sterling products and industrial components, while the other 7.5 wt.% is reserved for alloying elements^6^.

**Table 1:** Properties of the silver alloy powder.

Composition (%wt.)	Morphology	Bulk density (g/cm^3^)	Tap. density (g/cm^3^)	Size distribution (μm)		
				d_10_	d_50_	d_90_
92.5Ag, 7.5Cu	Spherical	10.40	5.9	16	25	39

### Parameter settings and preparation of sample by AM and casting

A Mysint100 SLM device was equipped with an active fiber laser (wavelength: 1070 nm, laser beam size: 0.030 mm, tuneable laser power: 0–200 W). The metal powder was layered with a rubber scraper. The distance between the bottom of the scraper and the previous layer was defined as the layer thickness (*h*). By spreading a thin layer over the previous layer, the laser selectively melted each layer of powder in the set area and welded it with the previous layer to build metallic parts. As shown in Fig. 11a, the main parameters included the laser power (*P*), scan speed (*v*_*l*_), layer thickness (*h*), hatch distance (*d*) and scan strategy (*s*), of which *d* refers to the distance between two adjacent scanning traces in the XY-plane. Meanwhile, the LED (Eq. (2)) was also introduced as a reference factor^8^.

2 $$\mathrm{LED}=\frac{P}{{v}_{l}\cdot h\cdot d}$$

The process parameters were adjusted to determine the LED range needed to achieve a high-volume density. Previous studies^21,73^ have shown that laser power plays a critical role in the influence of LED on material properties. The values of > 80 W and < 80 W are defined as high and low LED, respectively.

The hatch distance is calculated according to the overlap ratio (*Hr*) of the melt pool width (*w*) under different parameters, according to Eq. (3).

3 $$d=(1-Hr)\times w$$

According to Eq. (3), a higher overlap ratio (*Hr*) is associated with a smaller hatch distance (Fig. 11a). In this experiment, the overlap ratio (*Hr*) ranged from 30 to 70%.

Three types of models were prepared: a single-wall, cube and tensile rod. Their microstructures, morphologies, densities, phases, and mechanical properties were then characterized. The detailed parameters are listed in Fig. S3, Supplementary Information. As shown in Fig. 11b, the single-wall sample was formed by scanning a single-track layer-by-layer in the Z-axis, so the single-wall width was that of the melt pool. The cube samples can be considered as a parallel arrangement of single walls in the XY-direction (Fig. 11c). Analysis of single-wall samples served as a basis for analysis of the parameters and performance of the cube samples^21^. Compared to single-wall tests, which only involved three parameters (*P*, *v* and *h*), the cube and tensile tests required further consideration of parameters *d* and *s*. The scanning strategy (*s*) was designed by Materialise Magics software. The bi-offset scanning strategy included an angular-offset in the XY plane and a positional-offset on the next layer (Fig. 11d-h). Finally, the bi-offset scanning strategy forms meso-scale columnar “grains” along a < 001 > direction (Fig. 11h).

The parameter optimization process used to obtain high density was firstly based on analysis of the single-wall morphology, which obtained suitable parameters for laser power, scan speed and layer thickness. Through the cube test, two groups of parameters that produced the high-volume densities were selected as optimum, while one group of parameters that produced a lower volume density was used as reference. These three groups of parameters were mentioned repeatedly, so the samples prepared were named α, β, and γ, respectively (Fig. 4, 7, 9, 10). They were used to manufacture cube samples for the analysis of morphology, microstructure and density, as well as tensile rod samples for testing of mechanical properties. According to the ISO 6892–1-2009 standard, building directions of 90° were used to fabricate the tensile rods. A control group consisting of tensile rod samples of the same sizes and raw materials but prepared by casting were manufactured for comparison. Cast samples made of wax were place in a container of liquid plaster. Once the plaster set, the wax was molten in a furnace, and the remaining plaster became the mould. Then, molten Ag alloy (950 °C) was poured into this mould through a sprue gate and allowed to set. Finally, the mould was opened after 12 h using water injection^5^.

### Sectioning and sample preparation for characterization

Representative samples were prepared for characterization of morphology and defects by slicing them along the XY- and ZY-planes. Sections were ground and polished for SEM–EDS and XRD, using standard metallographic method up to 0.3 μm silica^21^. Then, 50 ml ammonia water, 50 ml H_2_O_2_ (3 vol.%) and 50 ml distilled water were used as an etchant to reveal the morphology and microstructure^21^. Representative sample preparation using argon ion polishing for EBSD.

### Materials characterization

Morphological and microstructural images were obtained by an optical microscope (LeicaM205; OM) and a scanning electron microscope equipped with an energy dispersive spectroscope (Hitachi-Su8010; SEM–EDS), respectively. The average grain size was measured from optical micrographs of each alloy using the linear intercept technique^44^. The IPF maps and pole figures were obtained by OXFORD C-Nano EBSD at 20 kV.

The Archimedes method was used to calculate the volume density^21^. The relative density was obtained by dividing the measured density by the theoretical density^21^. ImageJ 1.52 software was used to analyse the morphology and porosity based on colour thresholds^54^. It should be noted here that since different methods were used for volume density and porosity testing, led to the existence of incomplete agreement in the test results. This is due to the non-homogeneous of sample defects leading to errors in the porosity tested based on the cross-sectional area method. Therefore, on a macro-scale, the volume density tested based on Archimedes' principle is more representative of the densifications of a sample.

Elemental analysis of the samples was carried out using an energy dispersive spectroscope (EDS). Representative samples (orientation: Y-axis) were subjected to XRD diffraction (Bruker AXS D8-Focus diffractometer) to determine the phases and possible preferential crystallographic orientations induced by the process. The analyses were carried out with Cu-Kα radiation at 40 kV and 40 mA in the range 2θ = 20°–90°, using a step size of 0.02^74^.

### Mechanical testing

**Figure 12:**
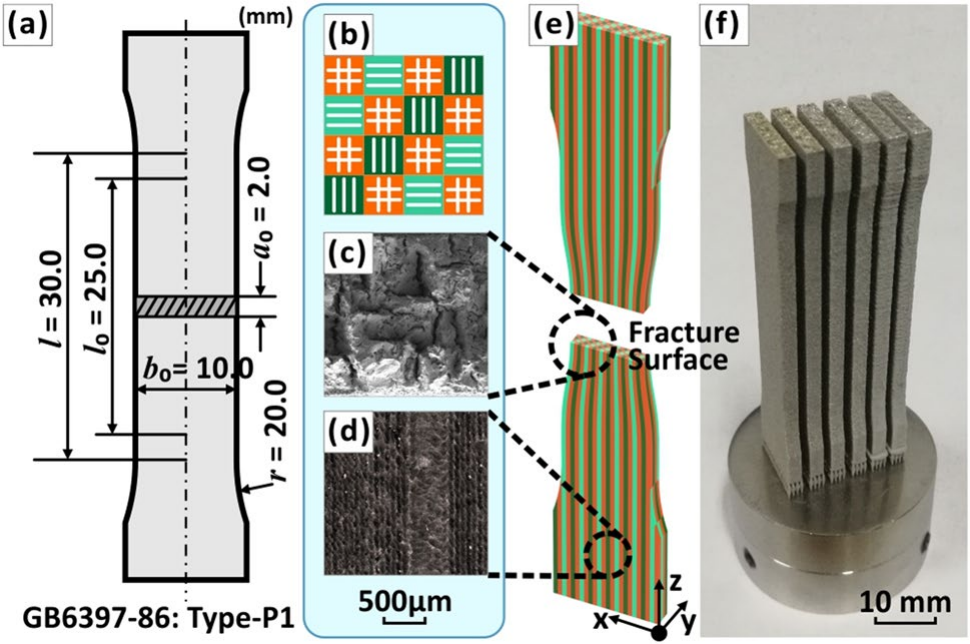
(**a**) Specimen dimension for metal tensile test^76^. (**b**) Schematic of the bi-offset scanning strategy. (**c**) a SEM image of fracture surface morphology. (**d**) an OM images of molten pool topography. (**e**) The relationship between the meso-scale “grain” and the directions of the tensile test. (**f**) an OM image of printed tensile test specimen.

Tensile specimen dimension was determined according to GB6397-86 standard (Fig. 12a). Tensile properties of the samples were obtained in accordance with ISO 6892-1-2009 standard, using a servo-electric CMT4304 frame equipped with a 30-kN load cell. Samples were clamped by the ends of the dog-bone-shaped samples. The extension rate was 2.0 mm/min and samples were loaded until fracture. All tests were performed at least three times for each condition and average value are reported^62,75^ (Fig. 12b–f).

### Simulation of solidification process, physical properties and AM process

Scheil-Gulliver solidification models, thermal conductivities, and cooling curves of the Ti-Cu and Ag–Cu alloys were simulated using JMatPro 7.0.0 DEMO software^54^. The *Q* values for the Ti alloys and Ag alloys were determined from the initial slope of Scheil-Gulliver solidification curve, given specifically by Eq. (4)^44^.

4 $$Q={\left(\frac{\partial \left({\Delta T}_{cs}\right)}{\partial {\Delta f}_{s}}\right)}_{{\Delta f}_{s}\to 0}$$

Rhinoceros 5.0 software was used to establish FEA 3D model for the AM process, while Simufact-Welding 6.0.0 software^64^ was used to simulate the AM process under bi-offset scanning strategy, as well as its temperature field, peak temperature, total deformation and equivalent plastic strain. For FEA simulations of the AM process related to different scanning strategies, see Video S1, Supplementary Information.

## Supplementary Information

Supplementary Information.
